# Left ureteral appendiceal interposition: Exercise caution and do not be mislead by postoperative radiological obstruction

**DOI:** 10.1590/S1677-5538.IBJU.2017.0295

**Published:** 2018

**Authors:** Aderivaldo Cabral Dias, Carlos Alberto Toledo Martinez, Maria Bianca Côrte, Marcus Vinicius Osorio Maroccolo

**Affiliations:** 1 Unidade de Urologia, Hospital de Base do Distrito Federal, Brasília, DF, Brasil; 2 Unidade de Proctologia, Hospital de Base do Distrito Federal, Brasília, DF, Brasil

## Abstract

Postoperative imaging after appendiceal ureteral interposition may be difficult to interpret, misguiding the urologist towards intervention. We present a case in which radiological obstruction was not endorsed by a 99TcDTPA nephrogram, with favorable outcome after conservative treatment.

## CASE PRESENTATION

**Figure 1:**
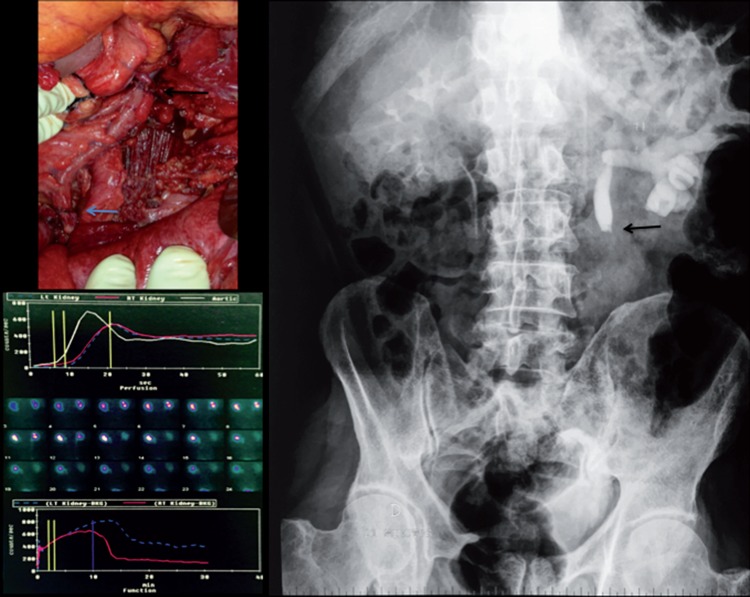
Upper Left: Intraoperative photograph showing proximal (black arrow) and distal ureteroappendiceal anastomoses (blue arrow). The mesoappendix can be seen on the medial aspect of the appendix; Right: Intravenous pyelogram 29 days after removal of the double-J stent showed left pyeloureterectasis and obstruction at the proximal ureteroappendiceal anastomosis (black arrow). Lower left: 99TcDTPA nephrogram displayed pyeloureteroectasis but adequate drainage of the left renal unit.

**Figure 2:**
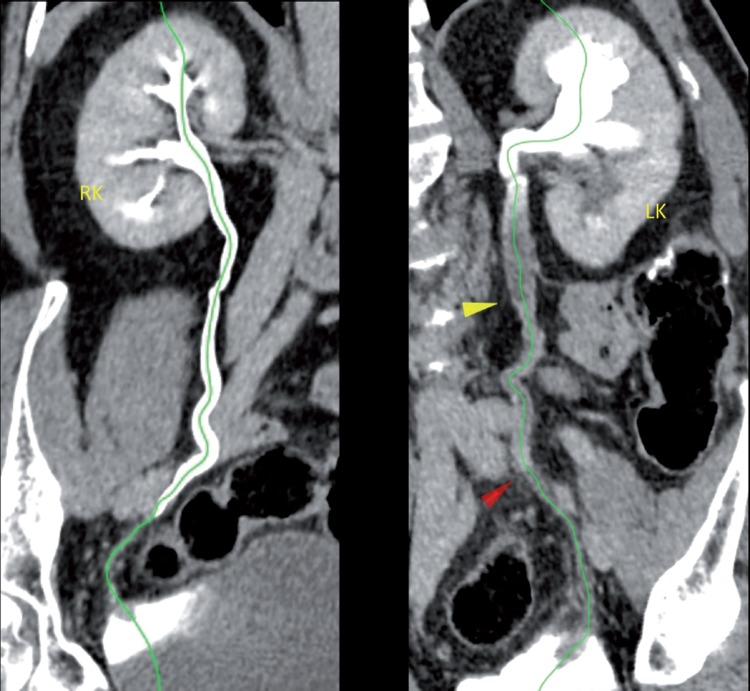
Curved multiplanar 3-dimensional reconstruction images of the right (left image) and left (right image) urinary tracts at the excretory phase two years after the operation: On the right, one observes the sites of the proximal anastomosis (yellow arrowhead), where the dilated, thin-walled left ureter joins the thick-walled appendix. The red arrowhead points to the distal appendicoureteral anastomosis. The green lines (3-dimensional Bézier paths) threads along both urinary tracts, from upper posterior calices to ureterovesical junctions. RK, right kidney; LK, left kidney

**Figure 3:**
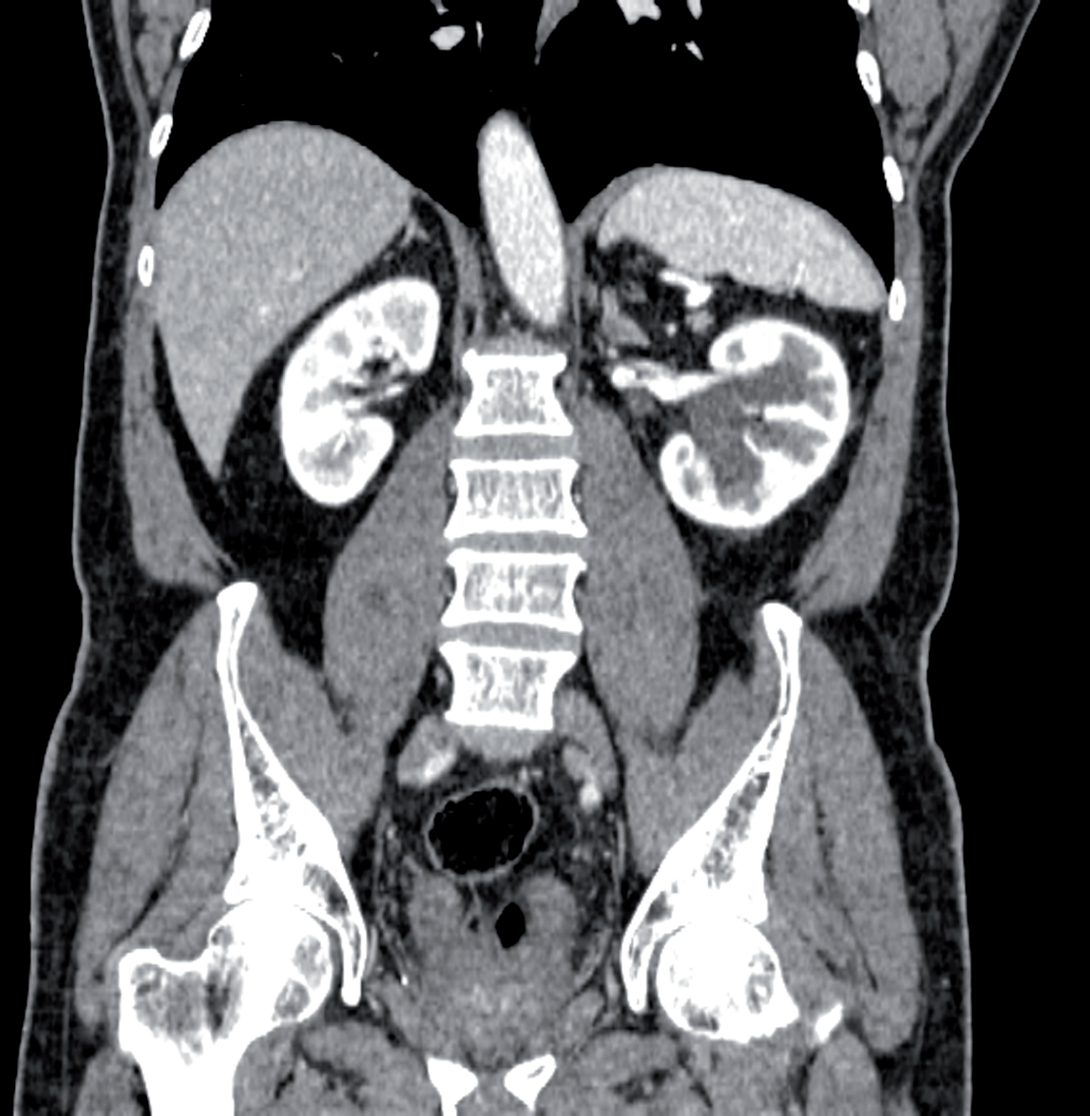
Coronal view of computed tomography at the nephrographic phase two years after the operation. One observes mild to moderate left pyeloectasis and good cortical contrast uptake.

Intraoperative consultation was requested by proctology. During left colectomy for adenocarcinoma, the left uppper-mid ureter of a 69-year old man was resected, leaving a 12cm gap. To spare the patient of another enteroenterostomy, antiperistaltic ureteroappendicoureterostomy was performed over a double-J stent (Figure-1, upper left). The patient was discharged from the hospital at the 17^th^ postoperative day (POD). We removed the double-J stent at the 53^th^ POD, and left pyeloureterectasis with obstruction at the proximal anastomosis was seen on an intravenous pyelogram performed at the 82^th^ POD (Figure-1, right). A 99TcDTPA nephrogram immediately followed, which showed adequate emptying (Figure-1, lower left). After 2 years the patient remains assymptomatic, with symmetric renal function (glomerular filtration rate: left=36.52, right=37.16mL/min/1.73m^2^). Computed tomography revealed mild-moderate left pyeloureterectasis, with good cortical uptake (Figure-2). Figure-3 displays both left and right urinary tracts as well as proximal and distal ureteroappendiceal anatomoses.

## DISCUSSION

The appendix can replace the left ureter via mobilization of the cecum and right colon (1-4). Due to the rarity of the procedure, and as previous case reports diverge regarding post-operative imaging routines, we opted for radiological surveillance according to our previous experience with ileal substitution of the ureter. Still, postoperative radiological abnormalities are not unexpected: There is a mismatch between the thin-walled ureter and thick-walled appendix, and ureteral peristalsis ceases at the ureteroappendiceal juncture.

Another possible explanation for the radiological aspect of obstruction we observed could be the choice of interposing the appendix in an antiperistaltic fashion. However, evidence has shown that antiperistaltic interposition does not hinder urine flow (5, 6). Since appendiceal peristalsis is not propulsive (7, 8), the interposed appendix behaves as a passive conduit, hence urine flows through the segment regardless of whether interposition is performed in an antiperistaltic or peristaltic fashion. Matter-of-fact, one could argue against peristaltic interposition, as it twists the mesoappendix, reducing its distal blood supply, which may cause leakage at the proximal anastomosis (5).

The astute urologist should be suspicious when challenged with incongruent clinic-radiological evidence of obstruction after appendiceal ureteral interposition, and proceed first with functional investigation.
